# Comparison of antimicrobial activities of natural essential oils and synthetic fragrances against selected environmental pathogens

**DOI:** 10.1016/j.biopen.2017.09.001

**Published:** 2017-09-13

**Authors:** Paula L. Vieira-Brock, Brent M. Vaughan, David L. Vollmer

**Affiliations:** 4Life Holdings, LLC, 9850 South 300 West, Sandy, UT 84070, USA

**Keywords:** Essential oils, Soap, Body spray, Air freshener

## Abstract

Plant essential oils (EOs) are known to inhibit the growth of bacteria and fungi. Whether these antimicrobial effects are comparable to synthetic household products is less clear. Furthermore, limited research is available on the potential additive effect of blending EOs. In this investigation, a new EO blend containing orange, patchouli, peppermint, and clary sage was compared to its individual single oils and to three household products–air freshener, liquid soap, and body spray–for their ability to inhibit the growth of *Staphylococcus aureus, Streptococcus pneumoniae, Pseudonomas aeruginosa, and Aspergillus brasiliensis* in the disc-diffusion assay. The new EO blend significantly inhibited the growth of the four microorganisms. The zones of inhibition of new EO blend were greater than the air freshener and similar to the liquid soap and body spray, with the exception of *Str. pneumoniae* in which the body spray provided greater inhibitory zone. The new EO blend and the single oils, with the exception of peppermint, equally inhibited the growth of *S. aureus* and *Str. pneumoniae* suggesting no additive effect. *P. aeruginosa* and *A. brasiliensis* showed variable susceptibility to all EOs except for no susceptibility to orange and limonene. No difference was found between (−) and (+)-limonene; whereas, (+)-menthol showed greater effect than (−)-menthol. In conclusion, blending the EO of orange, patchouli, peppermint, and clary sage was beneficial in inhibiting the growth of *S. aureus, Str. pneumoniae, P. aeruginosa, and A. brasiliensis* providing a natural antimicrobial fragrance option over synthetics fragrances used in soaps, body sprays, and air fresheners.

## Highlights

• A new essential oil blend inhibited the growth of four common environmental pathogens.
• The antimicrobial activity of a natural essential oil blend was similar or better to three synthetic products.
• Peppermint essential oil was the main ingredient in the essential oil blend that provided the antimicrobial activity.

## 1 Introduction

Plant essential oils (EOs) are volatile aromatic substances naturally produced by plants. Plant EOs have been used as natural fragrances by applying to the skin (functioning as a body spray) or diffusing into a room (functioning as an air freshener). In addition to the benefit of their natural pleasant scent, many plant EOs also have antimicrobial effects [1], [2], [3]. These two qualities of plant EOs foster their use as high quality products that are preferred over their synthetic counterparts that are either not natural or do not provide antimicrobial effects.

Despite extensive research on antimicrobial effects of single EOs [1], [2], [3] as well as on household products [4], [5], [6], limited research is available that compares the antimicrobial effects of natural EOs and synthetic household products [7], [8]. In one study, thyme EO and benzalkonium chloride were additive in reducing *S. aureus* in food [7]. Similar findings were reported by Shintre et al. (2006) in which lemon EO and benzalkonium chloride were synergistic in inhibiting the growth of *S. aureus* and *E. coli* [8]. Furthermore, it is unclear whether these antimicrobial effects of EOs remain once they are in a blend with other EOs.

A novel EO blend containing the EOs of orange, patchouli, peppermint, and clary sage, was formulated with the goal to achieve both, a high pleasant scent and an antimicrobial effect. In the current study, the primary objective was to evaluate the potential antimicrobial effect of this new EO blend against the common environment pathogens *P. aeruginosa*, *S. aureus*, *Str. pneumoniae*, and *A. brasiliensis*. A secondary objective was to compare the antimicrobial activity of the new EO blend with three household products – an air freshener, a body spray, and a liquid soap – that contained benzalkonium chloride, limonene and linalool, or neither. Lastly, in order to guide a potential improvement in formulation, studies were also performed to understand which component of the novel EO blend was responsible for any antimicrobial effect. For this set of experiments, the individual single EOs and major constituents contained in the new EO blend were assessed for their ability to inhibit the growth of the microorganisms.

## 2 Materials and methods

### 2.1 Test articles

Ten different samples were tested for their ability to inhibit the growth of the microorganisms described below. A new EO blend of the natural EOs of orange peel (*Citrus sinensis*), patchouli (*Pogostemon cablin*), peppermint leaf (*Mentha piperita*), and clary sage (*Salvia sclarea*), marketed as TForce™, was obtained from 4Life^®^ Holdings, LLC (Utah, USA). TForce also contains a small amount of proteins derived from cow colostrum and egg yolk that are not expected to contribute to any outcome measure assessed herein. The single natural EOs of orange, patchouli, peppermint, and clary sage were obtained from The Lebermuth Company, Inc. (Indiana, USA). The single constituents (S)(−)-limonene and (R)(+)-limonene were obtained from Sigma-Aldrich Corp (Missouri, USA). (+)-Menthol crystals were obtained from Alfa Aesar (Massachusetts, USA), and (−)-menthol crystals were obtained from Tokyo Chemical Industry Co., LTD (Oregon, USA). The synthetic cosmetics were purchased at regular stores: Glade spray air freshener aerosol Soho Social Citrus & Mimosa flower (lot number P365 686376) by Johnson & Son, Inc. (Wisconsin, USA), Noir Tease scented body mist spray (lot number 6323K2B2) by Victoria's Secret (Ohio, USA), and Soft on Skin Lemon & Verbena antibacterial liquid soap (lot number GN16242L607) by Lysol^®^ (New Jersey, USA).

### 2.2 Gas chromatography

The composition of the new EO blend was assessed according to ISO 11024 by Eurofins Scientific (Luxembourg City, Luxembourg). In brief, a gas chromatography coupled with a flame ionization detector (GC/FID) was used with the following analytical conditions: polar column: VB1; apolar column: DBWAX; dimensions: length = 60 m, diameter = 0.32 mm, stationary phase = 0.25 μm, vector gas = hydrogen; flow: 2 ml/min; division ratio: 1:100; split injection: 0.1  μl; injection temperature:250 °C; detection temperature: 260 °C; temperature programme: 60 °C for 15min, and 2 °C/min until 250 °C and 250 °C for 20 min.

### 2.3 Chiral analysis

The chiral method was adapted from the German standard §64 LFBG L00.00–106 and performed by Eurofins Scientific (Luxembourg City, Luxembourg). Briefly, analysis was conducted by first extracting the volatiles from samples and subsequently analyzing them by chiral gas chromatography mass spectrometry (GCMS). Extraction was performed by SDE (Simultaneous Solvent Distillation Extraction), followed by drying the extract using Na_2_SO_4_ and concentrating it by soft distillation with Vigreux column. GCMS separation was made using capillary column containing a chiral phase. The beta-cyclodextrins make it possible to separate the enantiomers and positional isomers. The recognition of the molecules was done by comparing the mass spectra and the retention time to a table of reference peaks obtained from standard materials. The enantiomeric proportions were calculated directly based on the area of the peaks.

### 2.4 Microorganisms used

The pathogens *Pseudonomas aeruginosa* ATCC 9027, *Staphylococcus aureus* ATCC 6538, *Streptococcus pneumoniae* ATCC 29514, and *Aspergillus brasiliensis* ATCC 16404 were obtained from and maintained in growth media at the Microbiology Division at Nelson Laboratories (Utah, USA). These microorganisms were selected for this study because they are common environmental organisms and their growths are inhibited by essential oils [1], [2], [3].

### 2.5 Antimicrobial activity

Bacteria were grown in soybean-casein digest broth at 35 °C for 24 h and diluted to 10^6^ CFU/mL with sterile distilled water. *A. brasiliensis* was grown in Sabouraud dextrose broth at 25 °C for 5 days and diluted with sterile distilled water to 10^4^–10^5^ CFU/mL. Diluted cultures were cooled and poured into each sterile plastic petri dish (85 mm diameter). An adaptation of Kirby-Bauer method (a.k.a., disc diffusion assay) was used [9]. Briefly, 50 μl (approximately 1 drop) of sample, water (negative control) or phenol (positive control), was added to a 6-mm sterile paper disc and placed on the center of each agar plate in direct contact with the medium. Menthol crystals were first diluted in hot water (95–110 F). A stock solution of 700 mg of menthol crystals into 1 mL of hot water was prepared to achieve the final testing concentration of 35 mg per 50 μl. The plates were covered with parafilm to prevent evaporation of the volatile substances. Inoculated plates were incubated at 35 °C for 24 h (bacteria) or 25 °C for 5 days (fungus).

### 2.6 Statistical analysis

The zone of inhibition was measured using calibrated calipers sensitive to 0.01 mm. When zones were absent, the value zero was entered. In cases of complete plate inhibition, the value considered was the size of the plate (85 mm). Statistical analyses were conducted using Statistica software (Dell, Oklahoma). The mean zone of inhibition ± standard deviation (SD) of triplicate measurements was analyzed using one-way ANOVA followed by Newman-Keuls post-hoc test for determination of significance among groups. Difference among groups were considered significant if the probability of type I error was <5% (*p* < 0.05).

## 3 Results

### 3.1 Description of ingredients in the test articles

The description of the ingredients in the tested products is presented in Table 1. The air freshener, body spray, and liquid soap contain several synthetic chemicals. The body spray contains synthetic limonene and linalool, which are constituents found in EOs including in the new EO blend. The liquid soap contains benzalkonium chloride which has shown to be an antimicrobial substance [4], [5], [6]. The extraction method and source of the single oils contained in the new EO blend are also presented in Table 1.

**Table 1:** Description of the ingredients of tested products.

Test article	Ingredients
New EO blend	essential oils of orange peel (*Citrus sinensis*), patchouli (*Pogostemon cablin*), peppermint leaf (*Mentha piperita*), and clary sage (*Salvia sclarea*)
Glade air freshener	water, isobutene, propane, sodium phosphate, sorbitan oleate, fragrance, propylene glycol, steartrimonium chloride
Noir Tease body spray	denatured alcohol, water, fragrance, propylene glycol, glycerin, butyl methoxydibenzoylmethane, ethylhexyl methoxycinnamate, ethylhexyl salicylate, chamomilla recutita flower extract, aloe barbadensis leaf extract, benzyl salicylate, coumarin, hydroxycitronellal, hydroxyisohexyl 3-cyclohexene carboxaldehyde, limonene, linalool, butylphenyl methylpropional, Red 40, Red 33, Yellow 5, Blue 1
Soft on skin liquid soap	benzalkonium chloride (0.10%), water, cetrimonium chloride, glycerin, PEG-150%), distearate, lauramine oxide, cocamide MEA, propylene glycol, citric acid, fragrance, tetrasodium EDTA, sodium chloride, hydrolyzed collagen, PPG-12-buteth-16, magnesium nitrate, butylene glycol, phenoxyethanol, methylchloroisothiazolinone, magnesium chloride, ethylhexyglycerin, methylisothiazolinone, FD&C yellow No.5, FD&C yellow No.6
Orange EO	essential oil of cold-pressed sweet oranges peel (*Citrus sinensis*) from Brazil
Patchouli EO	essential oil of steam-distilled patchouli (*Pogostemon cablin*) from Indonesia
Peppermint EO	essential oil of steam-redistilled peppermint (*Mentha piperita*) from U.S.A
Clary sage EO	essential oil of clary sage (*Salvia sclarea*) from France

Ingredients information were obtained either from the product label or from the product specification provided by the manufacturer.

### 3.2 Gas chromatography

GCMS analysis of new EO blend revealed the presence of 24 constituents (Table 2). Limonene was the major constituent followed by linalyl acetate, menthol, β-myrcene, and linalool. For ease comparison analysis, the composition of the single EOs is also provided in Table 2. These data obtained from the literature demonstrate that orange EO are mostly composed of limonene at levels similar to new EO blend. Clary sage EO's main constituent is linalyl acetate followed by linalool. These constituents were present in new EO blend at low amounts. Peppermint EO is mainly composed of menthol and menthone, and these constituents were also found in new EO blend. Finally, patchouli EO is mostly composed of patchoulol (patchouli alcohol), γ-guaiene and α-guaiene. These three constituents are present in the new EO blend, although at amounts lower than 1.00%.

**Table 2:** Composition of EOs obtained by GC/MS analysis.

New EO blend	limonene (84.50%), linalyl acetate (2.60%), menthol (1.90%), β-myrcene (1.60%), linalool (1.10%), menthone (0.95%), γ-guaiene (α-bulnesene) (0.76%), α-guaiene (0.64%), α-pinene (0.48%), patchouli alcohol (0.40%), α-patchoulene (0.39%), β-guaiene (seychellene) (0.31%), sabinene (0.30%), β-phellandren (0.28%), β-caryophyllene (0.27%), 1,8-cineole (eucalyptol) (0.23%), α-terpineol (0.22%), menthyl acetate (0.22%), neomenthol (0.16%), pulegone (0.15%), isomenthone (0.14%), delta-3-carene (0.12%), menthofuran (0.12%), pogostol (0.08%)
Orange EO Brazilian oranges	limonene (86.1–93.4%), β-myrcene (1.3–3.3%), β-bisabolene from Brazilian (0–1.5%), α-pinene (0.8–1.0%) [19]
Patchouli EO from Indonesian patchouli	patchouli alcohol (28.2–32.7%), γ-guaiene (α-bulnesene) (15.8–18.8%), α-guaiene (13.5–14.6%), β-guaiene (seychellene) (0–9.0%), γ-patchoulene (0–6.7%), α-patchoulene (4.5–5.7%), β-caryophyllene (3.1–4.2%), 1(10)-aromadendrene (0–3.7%), β-patchoulene (2.0–3.4%), pogostol (tr-2.4%), (−)-allo-aromadendrene (0–2.4%), γ-cadinene (0–2.4%) [19]
Peppermint EO from American peppermint	menthol (36.0–46.0%), menthone (15.0–25.0%), methyl acetate (3.0–6.5%), neomenthol (2.5–4.5%), 1,8-cineole (eucalyptol) (4.0–6.0%), menthofuran (1.5–6.0%), isomenthone (2.0–4.5%), β-pulegone (0.5–2.5%), limonene (1.0–2.5%), β-caryophyllene (1.0–2.5%), (E)-sabinene hydrate (0.5–2.3%) [19]
Clary sage EO from French clary sage	linalyl acetate (49.0–73.6%), linalool (9.0–16.0%), germacrene D (1.6–2.0%), β-caryophyllene (1.4–1.6%) [19]

### 3.3 Chiral analysis

As described above, the three main constituents of the new EO blend are limonene, linalyl acetate, and menthol. Chirality analysis was unable to differentiate (+) and (−) limonene; whereas, it was able to differentiate linalyl acetate and menthol. Results revealed that all menthol and linalyl acetate in the new EO blend are R isomers.

### 3.4 Antimicrobial activity

#### 3.4.1 Pseudonomas aeruginosa

Data presented in Table 3 reveal that new EO blend inhibits the growth of *P. aeruginosa* in comparison to water control (*p* < 0.05); although this antimicrobial activity was significantly lower than the positive control phenol (*p* < 0.05). The new EO blend inhibition of *P. aeruginosa*'s growth was similar to the inhibition provided by the body spray and liquid soap (*p* > 0.05). However, greater inhibition occurred with new EO blend, liquid soap, and body spray than with the air freshener (*p* < 0.05). Surprisingly, the single EO of orange, and its main constituent limonene (both isomers), did not inhibit the growth of *P. aeruginosa* (*p* > 0.05). On the other hand, the single EO of patchouli, peppermint, and clary sage inhibited the growth of *P. aeruginosa* to a similar or lesser extent than new EO blend (*p* < 0.05). However, despite growth inhibition provided by the peppermint oil, neither isomer of menthol inhibited *P. aeruginosa*'s growth (*p* > 0.05).

**Table 3:** Zone of inhibition (mm).

	*P. aeruginosa*	*S. aureus*	*Str. pneumoniae*	*A. brasiliensis*
New EO blend	15.92 ± 3.49 ^**a,b**^	22.62 ± 2.77 ^**a,d**^	21.22 ± 0.95 ^**a**^	22.14 ± 6.75 ^**a**^
Air freshener	0 ^**c**^	0 ^**b**^	13.25 ± 1.32 ^**b**^	0 ^**b**^
Body spray	18.44 ± 3.48 ^**a**^	27.36 ± 1.67 ^**a**^	35.02 ± 1.77 ^**c**^	16.76 ± 7.27 ^**a**^
Liquid soap	12.42 ± 1.21 ^**a,b**^	19.42 ± 1.44 ^**a,c,d**^	20.81 ± 0.05 ^**a**^	17.85 ± 2.37 ^**a**^
Orange EO	0 ^**c**^	17.88 ± 0.40 ^**a,c,d**^	23.64 ± 2.62 ^**a**^	0 ^**b**^
Patchouli EO	12.43 ± 0.75 ^**a,b**^	19.63 ± 1.14 ^**a,c,d**^	30.48 ± 0.34 ^**d**^	9.23 ± 0.44 ^**b**^
Peppermint EO	11.34 ± 1.18 ^**b**^	36.81 ± 4.89 ^**e**^	85 ± 0 ^f*^	74.16 ± 10.04 ^**d**^
Clary sage EO	2.57 ± 4.45 ^**c**^	22.70 ± 1.06 ^**a,d**^	43.49 ± 0.51 ^**e**^	32.14 ± 4.04 ^**c**^
(−)-Limonene	0 ^**c**^	13.10 ± 0.44 ^**a,c,d**^	18.33 ± 0.42 ^**a**^	0 ^**b**^
(+)-Limonene	0 ^**c**^	15.05 ± 1.00 ^**a,c,d**^	20.96 ± 0.56 ^**a**^	0 ^**b**^
(−)-Menthol	0 ^**c**^	11.57 ± 1.05 ^**c**^	20.71 ± 3.04 ^**a**^	5.41 ± 4.75 ^**b**^
(+)-Menthol	0 ^**c**^	14.68 ± 3.88 ^**d**^	33.41 ± 5.10 ^**c**^	17.19 ± 7.39 ^**a**^
Water	0 ^**c**^	0 ^**b**^	0 ^**b**^	0 ^**b**^
Phenol	55.78 ± 0.23 ^**d**^	54.67 ± 2.74 ^**f**^	48.51 ± 1.93 ^**e**^	66.47 ± 1.01 ^**e**^

Data expressed as mean ± S.D. of n = 3. Groups that do not share a common letter are significantly different (*p* < 0.05). Comparisons were made across different products of the same microorganism. No comparisons were made across the different microorganisms.

#### 3.4.2 Staphylococcus aureus

Similar to data on *P. aeruginosa*, the new EO blend inhibited the growth of *S. aureus* in comparison to water control (*p* < 0.05) (Table 3). Similar zones of inhibition were found with new EO blend, body spray and liquid soap (*p* > 0.05). In contrast, new EO blend had a greater zone of inhibition than the air freshener (*p* < 0.05). All of the single EOs in new EO blend, orange, patchouli, peppermint, and clary sage, significantly inhibited the growth of *S. aureus* when compared to water control (*p* < 0.05). However, a greater zone of inhibition was found with the peppermint EO (*p* < 0.05). Furthermore, both isomers of limonene oil significantly inhibited the growth of *S. aureus* when compared to water control (*p* < 0.05) with no difference between the (+) and (−) isomer. Both menthol isomers also inhibited the growth of *S. aureus*, with a greater effect of (+)-menthol, but smaller effect than peppermint EO (*p* < 0.05).

#### 3.4.3 Streptococcus pneunomiae

Likewise, the new EO blend inhibited the growth of the Gram-positive bacteria *Str. pneunomiae* (*p* < 0.05) (Table 3). Similar growth inhibition was found to the liquid soap. But, new EO blend and the liquid soap were more effective than the air freshener and less effective than the body spray (*p* < 0.05). The single EOs of orange and patchouli provided similar inhibition than the new EO blend. The peppermint and clary sage single EOs inhibited *Str. pneunomiae*'s growth to a greater extent than the other EOs (*p* < 0.05). Both, (+)-limonene and (−)-limonene also inhibited *Str. pneunomiae'*s growth to a similar extent than the other EOs. Lastly, both menthol isomers also inhibited the growth of *Str. pneumoniae*, with a greater effect of (+)-menthol, but smaller effect than peppermint EO (*p* < 0.05).

#### 3.4.4 Aspergillus brasiliensis

Lastly, the new EO blend also inhibited the growth of *A. brasiliensis* when compared to water control (*p* < 0.05) (Table 3). This antifungal effect of new EO blend was similar to the body spray and liquid soap, but greater than the air freshener (*p* < 0.05). Surprisingly, no inhibition of *A. brasiliensis* occurred with orange EO, (+)-limonene, and (−)-limonene. Patchouli EO presented a small zone of inhibition, lower than with the new EO blend (*p* < 0.05); whereas, peppermint and clary sage EOs provided a greater zone of inhibition than the new EO blend (*p* < 0.05). Only the (+)-menthol isomer inhibited *A. brasiliensis*'s growth, although this effect was significantly lower than the zone provided by peppermint EO (*p* < 0.05).

## 4 Discussion

The current study demonstrates that a new EO blend containing the EOs of orange, patchouli, peppermint, and clary sage, inhibits the growth of *P. aeruginosa, S. aureus*, *Str. pneumoniae*, and *A. brasiliensis* in the disc-diffusion assay. These antimicrobial effects of new EO blend were consistently greater than the growth inhibition provided by an air freshener, and similar to a body spray and liquid soap (with the exception of *Str. pneumoniae*, in which the body spray provided greater growth inhibition). Furthermore, the main ingredient in new EO blend, orange EO, does not appear to be the main contributor to new EO blend's effect against these microorganisms.

Previous studies have reported the antimicrobial effect of synthetic cosmetics or hygiene products. For example, benzalkonium chloride, the antibacterial ingredient in the liquid soap used in the current study, decreased the growth of Streptococcus when used with soap [4], lowered the rate of *P. aeruginosa* and *S. aureus* cultures in wounds of rats [6], and synergistically reduced *S. aureus* and *E. coli* when combined with lemon EO in an *in vitro* assay [8]. Similarly, limonene and linalool have shown antimicrobial activity against *S. aureus* and Aspergillus [2], [10], [11], [12]. As hypothesized, the current study also demonstrated that a liquid soap containing benzalkonium chloride and a body spray containing limonene and linalool reduced the growth of *P. aeruginosa, S. aureus*, *Str. pneumoniae*, and *A. brasiliensis*. To our knowledge, no antimicrobial ingredient is present in the common air freshener used in the current study in which results demonstrate no growth inhibition against *P. aeruginosa, S. aureus*, and *A. brasiliensis*. However, a small but significant antimicrobial effect was found against *Str. pneumoniae*.

Peppermint and patchouli EOs, but not orange and clary sage EOs, play a role in the inhibitory effect of the new EO blend against *P. aeruginosa*. In the current study, *P. aeruginosa* was inhibited by peppermint and patchouli EOs, but not by clary sage, orange or its constituent limonene suggesting that the patchouli and peppermint in the new EO blend are responsible for its effect against *P. aeruginosa*. Similar findings have been previously reported. Das et al. (2011) [1] found good inhibitory activity of patchouli oil against *P. aeruginosa* in the disc-diffusion assay. Furthermore, Sokovic et al. (2010) [2] reported good activity against *P. aeruginosa* with peppermint and menthol oils using the disc-diffusion assay. In contrast, no inhibition of *P. aeruginosa* was found in the disc-diffusion assay with clary sage or limonene oils [3], [13].

Similarly, the growth inhibition of *A. brasiliensis* by the new EO blend does not seem to be mediated by its main ingredient, orange EO. In the current study, *A. brasiliensis* was inhibited mainly by peppermint and clary sage EOs. The zone of inhibition provided by patchouli EO was small in comparison to the other oils and due to its content in the new EO blend being low (less than 1.00% of patchouli oil's constituents), patchouli oil is likely not playing a significant role in the inhibition of *A. brasiliensis*. Previous studies reported similar results with no inhibitory activity of *A. brasiliensis* with limonene or orange oils [3], [13], and good inhibitory activity with peppermint [14] and clary sage oils [3], although good growth inhibition of *A. brasiliensis* has also been reported with patchouli oil [15].

On the other hand, all of the oils inhibited the Gram-positive bacteria *S. aureus* and *Str. pneumoniae*. The new EO blend and its individual single oils, as well as the constituents limonene and menthol, inhibited the growth of *S. aureus* and *Str. pneumoniae*, although not to a similar extent, suggesting that all of the oils contributed to the antibacterial effect of the new EO blend. Particularly, *S. aureus* showed greater susceptibility to peppermint EO, and *Str. pneumoniae* showed greater susceptibility to peppermint and clary sage EOs, suggesting that these EOs might have played a greater role in the new EO blend's antimicrobial effect against these two bacteria. These data are in agreement with previous reports in which good antimicrobial effects against *S. aureus* and *Str. pneumoniae* were found with orange [2], [16], patchouli [1], [17], menthol [2], and clary sage [3] EOs.

Some additive or synergistic effect was found in blending the four single EOs. For example, the combination of the four EOs (i.e., new EO blend) provided greater antimicrobial effect than: orange EO alone (against *P. aeruginosa* and *A. brasiliensis*); patchouli EO alone (against *A. brasiliensis*); peppermint EO alone (against *P. aeruginosa*); and clary sage EO alone (against *P. aeruginosa*). Despite of the fact that some single EOs provided greater zones of inhibition than the new EO blend, only the new EO blend and the peppermint single EO inhibited all of the microorganisms tested. It is possible that the greater susceptibility of *S. aureus, Str. pneumoniae,* and *A. brasiliensis* to peppermint EO versus the new EO blend is due to the fact that the peppermint EO contains more of its main constituent menthol (e.g. 36.0–46.0% [19]) than the new EO blend (1.90%, Table 2). Thus, blending these single EOs provided greater benefit than the single EOs alone, but the new EO blend might be improved by reducing the amount of orange EO and increasing the amount of peppermint EO.

Despite significant differences in antimicrobial activity of the new EO blend and household products, the effect of dilution cannot be ruled out. In other words, the household products are diluted at a final concentration ready for consumers use; whereas, the EOs are concentrated and typically diluted before applied to skin or diffused. Thus, it can be speculated that the antimicrobial effects of the EOs presented herein occurred due to them being highly concentrated. However, the lack of antimicrobial activity of orange EO exemplifies that concentration is not the only factor for antimicrobial effects. Nevertheless, future studies are warranted to evaluate the effect of dilution on the EOs antimicrobial activity.

Lastly, no significant differences in antimicrobial activity were found between the (+) and (−)-limonene isomers, but (+)-menthol was more effective. These results are in accordance with Aggarwal et al. (2002) [18] in which both limonene isomers had similar activity against several bacteria and fungi, although other studies have found differences in the antimicrobial and physiological effects of (+) and (−) limonene [10], [19]. To our knowledge, this is the first study to report differences in the antimicrobial effect of (+) and (−)-menthol. (−)-Menthol is the major isomer found in nature [20]. The new EO blend contained exclusively (−)-menthol, which was found to be the lesser active isomer against *S. aureus*, *Str. pneumoniae*, and *A. brasiliensis*. We were unable to determine which limonene isomer is present in the new EO blend, but since no differences in antimicrobial activity were found between these two isomers, no relevant information would have been made.

## 5 Conclusion

The current study demonstrated that a blend of EOs of orange, patchouli, peppermint, and clary sage, provides greater growth inhibition of *P. aeruginosa*, *S. aureus*, *Str. pneumoniae*, and *A. brasiliensis* than an air freshener, and similar inhibition to a liquid soap, and a body spray (with the exception of *Str. pneumoniae*, in which the body spray provided greater growth inhibition). These antimicrobial effects of new EO blend were likely not provided by the orange essential oil, seen that orange essential oil and its main component limonene were not effective or less effective than the EO blend in inhibiting the four microorganisms. Blending these four essential oils provided a wider antimicrobial benefit than observed by its individual single oils. However, further investigation might elucidate whether reducing the amount of orange and increasing the amount of peppermint essential oil improve the antimicrobial effects of this blend.

## Abbreviations

EO.

## Conflict of interest

PVB, BV, and DV are currently employees of 4Life Holdings, LLC.

## Funding

This study was funded by 4Life Holdings, LLC.

## Authors' contributions

**PVB** designed the study, interpreted the data, wrote the manuscript and made substantial contribution to conception and data analysis. **BV** performed data analysis and made substantial contribution to conception, design and revision of the manuscript. **DV** conceptualized and designed the study, and made substantial contribution to data analysis and interpretation of data. All authors read and approved the final manuscript.
